# High frequency and long persistency of ballooning hepatocyte were associated with glucose intolerance in patients with severe obesity

**DOI:** 10.1038/s41598-021-94937-4

**Published:** 2021-07-28

**Authors:** Keisuke Kakisaka, Akira Sasaki, Akira Umemura, Haruka Nikai, Yuji Suzuki, Masao Nishiya, Tamotsu Sugai, Hiroyuki Nitta, Yasuhiro Takikawa

**Affiliations:** 1 Division of Hepatology, Department of Internal Medicine, Iwate Medical University School of Medicine, 1-1-1 Idaidori, Yahaba-cho, Iwate, 0283694 Japan; 2 Department of Surgery, Iwate Medical University School of Medicine, Iwate, Japan; 3 Department of Molecular Diagnostic Pathology, Iwate Medical University School of Medicine, Iwate, Japan

**Keywords:** Diseases, Gastroenterology

## Abstract

Nonalcoholic steatohepatitis (NASH) and glucose intolerance are associated with an increased risk of mortality in patients with severe obesity; however, whether histological findings of the liver are related to glucose intolerance in these patients remain unknown. Sixty-nine consecutive patients who underwent metabolic surgery between June 2008 and February 2020 were included; histological findings of the liver and laboratory data were analyzed. Twenty patients with biopsy-proven NASH were chronologically evaluated using sequential biopsies; data before metabolic surgery was considered as the baseline. Glucose intolerance—demonstrated by an increased area under the curve (AUC) for blood sugar (BS) during the 75-g oral glucose tolerance test—and increased homeostatic model assessment for insulin resistance (HOMA-IR) correlated with the grade of hepatocyte ballooning in patients. Patients with persistent ballooning at the follow-up biopsy had a higher HOMA-IR, high AUC for BS, and lower adiponectin level than those in patients in whom ballooning was eliminated, while there was no significant difference in body weight. We concluded that glucose intolerance was associated with the grade of hepatocyte ballooning; additionally, persistent hepatocyte ballooning sustained glucose intolerance, while elimination of hepatocyte ballooning improved the condition. Glucose intolerance may, thus, mediate balloon formation of the hepatocyte.

## Introduction

Severe obesity is one of the most serious public health concerns due to its globally increasing prevalence. Comorbidities associated with severe obesity—such as cardiovascular disease or type 2 diabetes mellitus (T2DM)—are increasingly becoming a cause of death^1,2^; thus, severe obesity should be considered a therapeutic target^3^. Bariatric surgery, such as laparoscopic sleeve gastrectomy (LSG), has been shown to be an effective method for achieving sustained weight loss^3^. As a result, patients with T2DM who undergo LSG can be maintained without any T2DM medications^4–6^. Bariatric surgery has been renamed “metabolic” surgery as it seems to be a functional cure that enables a return to healthy conditions^7^. Our previous study found several factors associated with T2DM remission in patients who underwent LSG^5^; by contrast, some patients exhibited persistent glucose intolerance or T2DM despite weight reduction being achieved through metabolic surgery^5^.

Glucose intolerance and insulin resistance result from impaired glucose metabolism; the main organs responsible are the liver, skeletal muscle, and adipose tissue^8^. Still, the mechanism by which the liver affects glucose metabolism following weight reduction by metabolic surgery remains unknown in patients with persistent glucose intolerance or T2DM. Histological findings of the liver are associated with present metabolic status and may predict the clinical course after metabolic surgery since the liver is the central organ of metabolism^9^. Histological findings associated with a functional cure for glucose metabolism or those associated with persistent glucose intolerance and/or insulin resistance can, therefore, be used to establish appropriate therapeutic strategies for patients after metabolic surgery.

Nonalcoholic fatty liver disease (NAFLD) is also a known comorbidity of severe obesity^10^. Following the successful eradication of hepatitis C by direct-acting antivirals, and with the increasing prevalence of obesity worldwide, nonalcoholic steatohepatitis (NASH)—a severe form of NAFLD—is thought to be the leading cause of liver cirrhosis and hepatocellular carcinoma^11^. Importantly, although metabolic surgery improves fibrosis, there is no agent to cure NASH^12^; therefore, metabolic surgery is one of the most promising treatments for patients with severe obesity-associated NASH. The liver plays a central role in protein synthesis and detoxification, as well as metabolism; therefore, the pathophysiology of NASH is affected by several organs, such as the gastrointestinal tract, visceral adipose tissue (VAT), subcutaneous adipose tissue (SAT), pancreas, central nervous system, and peripheral nervous system^13^. The hypothetical pathophysiology of NASH is, thus, complex^13^.

Insulin resistance refers to the impaired blood glucose-lowering effect of insulin^14^. It is a forerunner of T2DM and is often found in patients with obesity but without significant findings of T2DM^15^; therefore, insulin resistance is considered an antecedent condition in the pathophysiology of T2DM in patients with obesity. Insulin resistance has been shown to occur in subjects with obesity with a preponderance of VAT and NAFLD (ectopic fat accumulation in the liver)^16–18^. Weight reduction results in a decrease in the quantity of VAT, as well as inflammation of VAT, resulting in improved insulin resistance. Regarding the pathophysiology of NAFLD, histological findings (inflammation, steatosis, and hepatocyte ballooning) have been evaluated to estimate disease activity^19^. In addition to VAT, we hypothesized that histological findings of NAFLD are associated with insulin resistance in patients with severe obesity. As this is not yet proven, the primary aim of the present study was to determine whether histological findings of NAFLD are associated with glucose intolerance and/or insulin resistance in patients with severe obesity. To satisfy the primary aim, we evaluated the histological findings and impaired glucose metabolism of patients with severe obesity who underwent LSG.

## Results

### Patients

**Figure 1:**
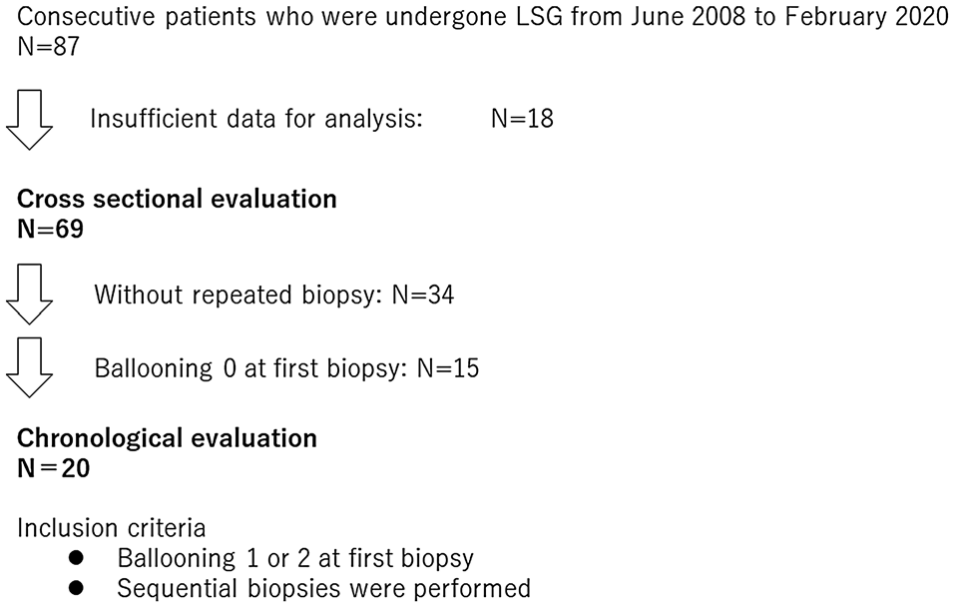
Patient flow diagram of the present study.

Between June 2008 and February 2020, 87 patients who underwent LSG were prospectively enrolled in the database (Fig. 1). Of these 87 patients, 18 were excluded due to a lack of data; 69 patients were analyzed regarding the relationship between glucose metabolic markers and histological findings. Hepatocyte ballooning was identified as a histological finding associated with insulin resistance; the results are presented in the following section. Thus, we focused on the serial changes in hepatocyte ballooning and glucose metabolism. Of the 69 patients, 35 underwent sequential biopsies; 20 of 35 patients exhibited grade 1 or 2 hepatocyte ballooning at the first biopsy. To determine the clinical impact of histological changes on glucose metabolism or anthropometric data, the patients were divided to two groups according to the “elimination” or “persistence” of hepatocyte ballooning (based on the presence or absence of hepatocyte ballooning at the indicated time of the follow-up biopsy).

### Hepatocyte ballooning was identified as a factor associated with insulin resistance

To determine the histological findings associated with insulin resistance and glucose intolerance, we performed linear regression analysis using the HOMA-IR, HOMA-β, AUC for BS, and AUC for IRI as the objective variables and the histological findings of NAS as explanatory variables (Table 1). During HOMA analysis, hepatocyte ballooning was the only factor associated with HOMA-IR; no factors were associated with HOMA-β. During AUC analysis for BS, liver fat and hepatocyte ballooning were negatively and positively related, respectively.

**Table 1:** Linear regression analysis of glucose metabolic markers using histological findings of the liver in patients who underwent metabolic surgery.

Objective variable	Explanatory variables	b	95% C.I		p value	
HOMA-IR	Steatosis	− 0.310	− 1.270	0.639	0.5110	
	Inflammation	0.261	− 0.809	1.332	0.6275	
	Hepatocyte ballooning	1.468	0.432	2.503	0.0062	*
HOMA-b	Steatosis	5.892	− 32.404	44.188	0.7596	
	Inflammation	10.291	− 32.668	53.250	0.6340	
	Hepatocyte ballooning	− 3.287	− 44.817	38.244	0.8749	
AUC_BS	Steatosis	− 2233.8	− 4025.9	− 441.8	0.0154	*
	Inflammation	1050.9	− 959.4	3061.3	0.3003	
	Hepatocyte ballooning	3207.4	1264.0	5150.9	0.0016	*
AUC_IRI	Steatosis	517.4	− 1738.8	2773.7	0.6485	
	Inflammation	1450.3	− 1080.7	3981.3	0.2567	
	Hepatocyte ballooning	391.4	− 2055.4	2838.3	0.7504	

*HOMA* homeostasis model assessment, *IR* insulin resistance; *AUC* area under curve; *BS* plasma sugar; *IRI* serum insulin.

### Elevated levels of aspartate aminotransferase and increased subcutaneous adipose tissue were found in patients with hepatocyte ballooning

In the above results, patients with a higher grade of hepatocyte ballooning were found to exhibit higher HOMA-IR results, indicating that the patients exhibited insulin resistance. Although there was no reasonable mechanism to elucidate why a lower grade of liver fat was associated with a high AUC for BS, liver fat was not associated with HOMA-IR; therefore, hepatocyte ballooning was considered a factor associated with insulin resistance, and the patients were further classified according to the grade of hepatocyte ballooning. Anthropometric and laboratory data were compared among the three groups with hepatocyte ballooning. AST and SAT at the baseline were significantly higher in patients with grade 2 than in those with grade 0 hepatocyte ballooning (Table 2); however, these differences were not observed at the first visit. In addition, the HOMA-IR and AUC for BS were significantly higher in the group with grade 2 ballooning (Table 3).

**Table 2:** Comparison of anthropometric and laboratory data among three groups with different ballooning grades.

	Ballooning 0 (n = 33)		Ballooning 1 (n = 18)		Ballooning 2 (n = 18)		
	Mean	SD	Mean	SD	Mean	SD	
Age	44.2	10.7	46.0	14.5	42.3	13.6	
Sex (female)	12		9		11		
**First visit**							
BMI (kg/m^2^)	42.9	5.8	43.6	8.5	42.8	4.2	
BW (kg)	119.8	16.6	121.3	29.2	115.3	21.8	
L/S ratio	0.90	0.22	0.78	0.30	0.69	0.26	p = 0.0258*
Total AT (cm^2^)	775.0	140.9	810.9	145.2	820.7	163.1	
SAT (cm^2^)	528.0	142.0	530.5	109.9	548.1	141.1	
VAT (cm^2^)	255.7	72.1	290.5	115.9	272.6	71.0	
**Baseline**							
DM	19		9		16		p = 0.0213* p = 0.0113***
Insulin preparation	10		2		2		
Oral medication for DM	14		7		12		
BMI (kg/m^2^)	38.0	4.0	39.5	4.8	39.6	4.2	
BW (kg)	106.0	13.7	109.6	20.5	106.7	21.5	
L/S ratio	1.14	0.23	1.01	0.22	0.85	0.31	p = 0.0043*
Total AT (cm^2^)	681.4	132.7	788.0	151.4	753.3	132.9	
SAT (cm^2^)	457.7	120.0	514.9	100.1	515.4	121.4	p = 0.0306**
VAT (cm^2^)	223.8	74.9	265.4	83.2	238.0	69.7	
**Laboratory data**							
AST (U/L)	35	30	39	26	66	45	p = 0.0399*
ALT (U/L)	52	52	60	53	85	60	
gGT (U/L)	51	39	49	35	77	60	
Alb (mg/dL)	4.3	0.4	4.4	0.4	4.3	0.3	
T-Bil (mg/dL)	0.8	0.4	0.8	0.3	0.7	0.3	
D-Bil (mg/dL)	0.2	0.2	0.2	0.1	0.2	0.1	
BUN (mg/dL)	16	6	15	6	13	3	
Cre (mg/dL)	0.86	0.39	0.80	0.35	1.15	2.21	
TC (mg/dL)	196	37	186	33	184	49	
TG (mg/dL)	147	90	155	96	163	125	
LDL-C (mg/dL)	127	30	113	31	117	33	
HDL-C (mg/dL)	43	10	48	14	44	12	
Plt (10^3^/mL)	252	53	258	68	265	80	
HbA1c (%)	6.1	1.3	6.1	1.2	6.8	1.0	
BS (mg/dL)	93.9	17.1	103.6	23.3	111.0	24.8	p = 0.0087*
IRI (ng/mL)	12.9	8.3	13.3	7.9	21.3	11.2	p = 0.0028*
Adiponectin (mg/mL)	1.91	1.05	2.35	1.59	1.97	1.15	

*BMI* body mass index; *BW* body weight; *L/S ratio* ratio of liver to spleen; *AT* adipose tissue; *SAT* subcutaneous adipose tissue; *VAT* visceral adipose tissue; *DM* diabetes mellitus; *AST* aspartate aminotransferase; *ALT* alanine aminotransferase; *gGT* gamma glutamyl transpeptidase; *Alb* albumin; *T-Bil* total bilirubin; *D-Bil* direct bilirubin; *BUN* blood urea nitrogen; *Cre* creatinine; *TC* total cholesterol; *TG* triglyceride; *LDL-C* low density lipoprotein-cholesterol; *HDL-C* high density lipoprotein-cholesterol; *Plt* platelet count; *HbA1c* hemoglobin A1c and IRI, serum insulin.

* and ** indicates statistical significance.

* was for ballooning grades 0 vs. 2, ** for grades 0 vs. 1 and *** for grades 1 vs. 2.

**Table 3:** Comparison of glucose metabolic markers among three groups with different ballooning grades.

	Ballooning 0 (n = 33)		Ballooning 1 (n = 18)		Ballooning 2 (n = 18)		
	Mean	SD	Mean	SD	Mean	SD	
AUC_IRI (min*ng/mL)	9604	7677	9263	5220	12,382	6978	
AUC_BS (min*mg/dL)	20,272	4546	21,343	6103	25,624	7071	*p = 0.0203
HOMA-b	174.9	131.3	143.5	82.1	189.0	114.3	
HOMA-IR	3.06	2.24	3.46	2.34	6.08	4.17	*p = 0.0237

*HOMA* homeostasis model assessment, *IR* insulin resistance; *AUC* area under curve; *BS* plasma sugar; *IRI* serum insulin.

*Indicates a statistical significance between ballooning grades 0 and 2.

### Persistent hepatocyte ballooning was observed in patients with sustained insulin resistance, a high AUC for BS, and low adiponectin after LSG

**Figure 2:**
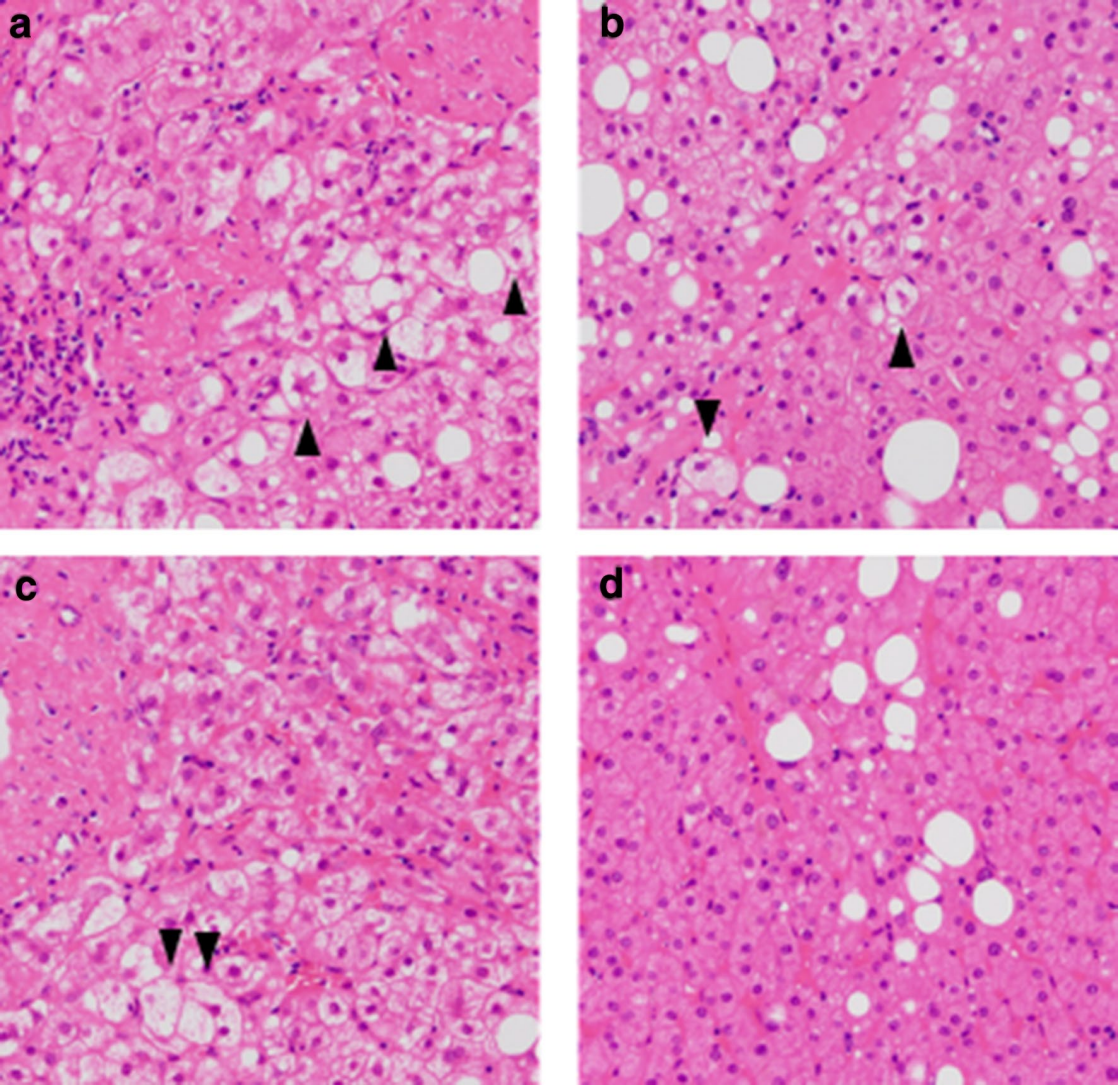
Histological images indicating hepatocyte ballooning. Histological images of liver biopsies using hematoxylin–eosin staining in one high-power field. Histological images of hepatocyte ballooning at the baseline in both the elimination (**a**) and persistent groups (**b**), and at indicated times in the persistent group (**c**) are presented; the arrowhead indicates hepatocyte ballooning. Liver histology at the indicated time in the elimination group is presented for comparison (**d**).

To determine the significance of hepatocyte ballooning in the pathophysiology of NAFLD, chronological changes in hepatocyte ballooning and serial changes in anthropometric and laboratory data were evaluated. Of the 35 patients who underwent sequential liver biopsies, 15 with grade 0 hepatocyte ballooning at the baseline exhibited no changes in the grade of hepatocyte ballooning. Of the remaining 20 patients, 13 had grade 0 hepatocyte ballooning at the indicated time points, while 7 patients exhibited grade 1 or 2 ballooning; thus, 13 patients were classified into the elimination group, and 7 into the persistence group (Fig. 2). BS, AUC for BS, IRI, and HOMA-IR at the baseline were significantly higher in the persistence group (Table 4); however, there was no difference in adiponectin, SAT, and VAT among the groups. When comparing the data at the indicated time points between the two groups, BS, AUC for BS, IRI, and HOMA-IR were significantly higher in the persistence group. Furthermore, although the adiponectin level and reduction of BW were significantly lower in the persistence group, there were no significant differences in SAT and VAT between the two groups.

**Table 4:** Comparison of anthropometric and glucose metabolic markers between the balloon elimination and persistence groups.

	Elimination (n = 13)		Persistence (n = 7)		
	Median	Rage	Median	Rage	
Age	43 (21–65)		58 (21–64)		
SEX(F:M)	(6:7)		(6:1)		
Follow-up duration	6M (7), 1y (2), 2y (3), 3y (1)		6M (5), 1y (1), 2y (1)		
Ballooning grade at first biopsy	1(9), 2(4)		1(2), 2(5)		
**Baseline**					
BW	118.6	(81.4–159.2)	88.0	(67.2–109.6)	
AUC_BS (min*mg/dL)	18,240.0	(13,275.0–31,455.0)	29,475.0	(20,685.0–39,930.0)	p = 0.0258
AUC_IRI (min*ng/mL)	9712.0	(3390.0–21,091.5)	9336.0	(6202.5–27,156.0)	
HOMA-b	139.4	(32.4–421.7)	116.0	(83.8–298.4)	
HOMA-IR	3.104	(0.952–6.480)	7.856	(2.836–9.979)	p = 0.0021
BS (mg/dL)	87	(77–142)	119	(99–167)	p = 0.0139
IRI (ng/mL)	12.7	(4.7–24.6)	23.4	(11.6–31.5)	p = 0.0475
Adiponectin	1.83	(0.94–5.64)	1.33	(0.49–2.37)	
SAT (cm^2^)	547.1	(255.3–743.6)	550.4	(436.8–629.2)	
VAT (cm^2^)	272.0	(132.6–385.3)	235.0	(127.9–311.3)	
**Post operation**					
BW (kg)	83.0	(60.5–117.8)	79.0	(67.2–93.5)	
Reduction of BW (kg)	21.8	(12.8–51.3)	14.6	(4.8–20.5)	p = 0.0264
AUC_BS (min*mg/dL)	17,085.0	(11,970.0–34,740.0)	22,965.0	(19,800.0–33,075.0)	p = 0.0249
AUC_IRI (min*ng/mL)	5418.0	(3013.5–9291.0)	8404.5	(6453.0–55,813.5)	
HOMA-b	147.0	(19.6–265.5)	100.8	(59.1–193.5)	
HOMA-IR	1.296	(0.356–2.572)	4.035	(1.034–7.191)	p = 0.0224
BS (mg/dL)	84	(72–121)	103	(91–134)	p = 0.0048
IRI (ng/mL)	7.0	(2.0–12.4)	13.9	(4.6–23.3)	p = 0.0157
Adiponectin (mg/mL)	4.19	(2.19–9.16)	2.52	(0.99–2.52)	p = 0.0200
SAT (cm^2^)	325.9	(111.3–637.3)	420.7	(325.4–497.1)	
VAT (cm^2^)	123.1	(68.7–313.5)	165.4	(93.4–205.6)	

*BW* body weight; *AUC* area under curve; *BS* blood sugar; *IRI* serum insulin; *HOMA* homeostasis model assessment; *IR* insulin resistance; *BS* plasma sugar; subcutaneous adipose tissue and VAT, visceral adipose tissue.

## Discussion

The present study revealed that hepatocyte ballooning was associated with insulin resistance in patients with severe obesity at baseline. Additionally, patients with persistent hepatocyte ballooning after metabolic surgery exhibited insulin resistance, while the patients who exhibited elimination of hepatocyte ballooning after the surgery demonstrated improved insulin resistance.

Weight reduction is the first step in the establishment of treatment for patients with NAFLD who are overweight^20^. Clinical guidelines for NAFLD by American Association for the Study of Liver Diseases. recommend a 10% weight reduction to attenuate inflammation in the NASH liver^20^. As nutritional intervention before metabolic surgery resulted in an approximate 10% weight reduction in most patients in our institute, fat deposition decreased or was absent during the liver biopsy at baseline in some of the patients who achieved weight reduction^12^. In contrast, fibrosis was found at the baseline, and this gradually resolved 1 or 2 years following metabolic surgery^12^. Previous data suggested that steatosis could be resolved through short-term action, while resolution of fibrosis required long-term action^12^. To understand the beneficial effects of weight reduction, data regarding serial changes in metabolic status and histological findings in patients who have undergone LSG are believed to be useful. At the baseline, hepatocyte ballooning was associated with insulin resistance in patients with severe obesity; additionally, the persistence of hepatocyte ballooning after metabolic surgery was also associated with insulin resistance, although most patients who underwent sequential liver biopsies exhibited decreased weight, SAT, and VAT. Thus, hepatocyte ballooning is considered a key finding regarding insulin resistance in the liver. This finding was proved by another research of observational study for NASH patients with or without insulin resistance^9^.

In the FLIP algorithm, Brunt grade, and Matteoni’s classification, hepatocyte ballooning is the histological hallmark of NASH^21^. Although the pathogenesis of hepatocyte ballooning remains unknown, balloon formation is considered a result of hepatocyte injury. Hepatocyte ballooning and dying hepatocytes with sustained injury express fibrogenic signals, such as hedgehog signaling^22,23^. Intriguingly, increased expression of Sonic hedgehog in the liver can predict future NASH^24^. According to these findings, Sonic hedgehog expression due to liver injury promotes balloon formation in hepatocytes^23^. Previous papers reported that hepatocyte balloon formation was associated with an impaired cell death pathway under lipotoxicity in an in vitro study^25^, while the presence of hepatocyte ballooning was a predicted marker for the future progression of liver fibrosis^26^; therefore, hepatocyte ballooning may be involved in the pathophysiology of NASH.

Adiponectin may be associated with hepatocyte ballooning. In this study, the persistence group showed lower levels of adiponectin than the elimination group at the indicated time of sequential liver biopsy after surgery. A previous study reported that adiponectin levels were lower in patients with NAFLD than in controls^27^. While low levels of adiponectin in patients with NAFLD was reported to impair fatty acid metabolism and promote a chronic inflammatory state in the liver^27^. Furthermore, reduced adiponectin induces steatohepatitis via impaired mitochondrial biogenesis^28^. Autophagy, which plays a role in the quality control of mitochondria^29^, was found to be impaired in both the NASH liver^30^ and during hepatocyte ballooning^25^. Overall, impaired mitochondrial biogenesis due to low adiponectin levels and disruption of mitochondrial quality control due to impaired autophagy may contribute to the pathophysiology of NASH^25^.

The present study demonstrated a relationship between hepatocyte ballooning and insulin resistance. The term “insulin resistance” should carefully used as impaired insulin sensitization occurs in the liver, as well as in skeletal muscle and adipocytes; therefore, we could not determine which organ induced insulin resistance in the present study. Additionally, we could not ascertain whether the mechanism of insulin resistance remained the same between the baseline and indicated time of sequential biopsy. Based on speculation, we mentioned that insulin resistance occurred in the liver of the persistence group after surgery. Since there were no significant differences among the elimination and persistence groups regarding BW, SAT, and VAT, skeletal muscle mass was thought to be similar between the two groups; thus, we considered that the liver may have contributed to insulin resistance in the persistence group. Based on this hypothesis, the mechanism and functional analysis of hepatocyte ballooning are crucial to understanding the pathophysiology of NASH.

There were several limitations to this study; first, the number of patients included was small. However, the study was performed as an exploratory analysis; therefore, a sample size could not be calculated before analysis. Additionally, the treatment conditions were relatively homogenous. As the intervention employed in the present study was LSG, the onset of the intervention’s effect was clear throughout the observational period. In addition, weight reduction—the main effect of LSG—was induced in most patients. Although the multiple hit theory—which is a considerable pathophysiology of NASH—was complicated, the present study primarily focused on weight reduction in patients with NASH. Second, the patients in the present study may not reflect the general population with NAFLD as we included those with severe obesity. In Asia, severe obesity—defined as a BMI > 35 kg/m^2^—is relatively rare^1^; therefore, results from patients with severe obesity may be unrelated to the pathophysiology of NASH in patients without a BMI > 35 kg/m^2^. Nevertheless, a recent study reported histological similarities, such as fibrosis, between patients with NASH both with and without obesity^31^. Further studies that focus on the relationship between hepatocyte ballooning and insulin resistance in patients with NASH who are not severely obese are required to confirm the results of the present study. Finally, the characteristics of ballooning hepatocytes were not evaluated. As mentioned above, several specific characteristics—such as the accumulation of ubiquitin and absence of keratin 8/18 double staining—have been reported in ballooning hepatocytes^32^. We were unable to confirm these histological characteristics due to the absence of some liver biopsy samples; thus, we did not perform additional histological examinations. Noninvasive parameters for hepatocyte ballooning were recently proposed^33,34^, and using these parameters, the clinical significance of hepatocyte ballooning for insulin resistance may be evaluated. We aim to directly evaluate the relationship between specific characteristics of hepatocyte ballooning and the clinical course after LSG in future studies. Still, as the study was performed as an exploratory analysis, future, confirmatory analysis is required to validate the results of the present study.

## Patients and methods

### Patients

Patients with severe obesity who underwent LSG between June 2008 and February 2020 were consecutively registered in this study. All patients satisfied the following indications for metabolic surgery: between 18 and 65 years of age, severe obesity (body mass index [BMI] > 35 kg/m^2^), and at least one comorbidity with resistance to medication (hypertension, T2DM, dyslipidemia, and obstructive sleep apnea). BMI was expressed as body weight (kg) divided by height (m) squared. The following data were collected at the first visit (within 1 week before surgery), considered as the baseline, as well as at 6, 12, 24, and 36 months postsurgery: albumin (Alb), alanine aminotransferase (ALT), aspartate aminotransferase (AST), gamma glutamyl transpeptidase (γGT), hemoglobin A1c (HbA1c), high-density lipoprotein cholesterol (HDL-C), low-density lipoprotein cholesterol (LDL-C), platelet count (Plt), total cholesterol (TC), and triglyceride (TG). In addition, the quantity of VAT and SAT at the level of the navel, as well as the liver-to-spleen (L/S) ratio, were measured with computed tomography using SYNAPSE VINCENT imaging software (Fujifilm, Tokyo, Japan).

All study protocols were approved by the Institutional Review Board of Iwate Medical University (H27-47). The study was performed in accordance with the relevant guidelines and regulations, and informed consent was obtained from all participants.

### 75-g OGTT

A 75-g oral glucose tolerance test (OGTT)—with blood sugar (BS) and serum insulin (IRI) measurements at 0, 30, 60, 90, and 120 min—was performed to calculate the following parameters: the homeostatic model assessment-estimated insulin resistance (HOMA-IR), and the homeostasis model assessment for β-cell function (HOMA-β). Each parameter was calculated using the following formula:

- HOMA-IR: (IRI at 0 min µU/mL) × [BS at 0 min. (mg/dL)]/405
- HOMA-β: (360 × IRI at 0 min µU/mL)/ [BS at 0 min. (mg/dL) − 63]

To evaluate serial changes in BS or IRI during the 75-g OGTT, the area under the curve (AUC) was calculated; results were presented as the AUC for BS and AUC for IRI, respectively. To perform the 75-g OGTT, all patients with T2DM were subjected to glycemic control using a rapid-acting insulin analog one day before the examination to exclude the influences of long-acting insulin analogs.

### NAFLD activity score (NAS)

All samples were evaluated in a blinded manner by two senior pathologists, who scored inflammation, steatosis, and ballooning using NASs^35^. After unmatched results were discussed between the two pathologists without clinical information, reports with final scores were provided to the clinicians, and each score in the report was recorded in the database. To evaluate serial changes in hepatocyte ballooning, patients with improved ballooning grades across sequential biopsies were classified into the “elimination” group, while the patients who exhibited grade 1/2 sequential biopsies were classified into the “persistence” group. In the elimination group, the time point for evaluation was defined by the first time an improvement to grade 0 was observed, while in the persistence group, the time point for evaluation was the last time to confirm grade 1/2 hepatocyte ballooning; these time points were indicated as the postoperation points.

### Statistical analysis

The primary endpoints of this study were the histological findings of the liver associated with glucose intolerance in patients with severe obesity and NAFLD. Data are presented as mean values with standard deviation, or median with range (minimum–maximum). All analyses were performed using SPSS version 17.0 (SPSS Inc., Chicago, IL, USA) or JMP Pro 13 (SAS Institute, Cary, NC, USA). The Chi-squared test, Student’s t-test, Wilcoxon/Kruskal–Wallis test, and Mann–Whitney U test were used to evaluate the statistical significance of the results, and the Bonferroni method was used for post-hoc analysis after analysis of variance; a two-sided p-value < 0.05 was considered statistically significant. Linear logistic regression analysis was performed to identify the histological factors associated with the indicated variables. Histological findings according to the NAS (steatosis [0–3], lobular inflammation [0–3], and hepatocyte ballooning [0–2]) were used as explanatory variables. The contribution of explanatory variables to the objective variable was expressed as the odds ratio.
